# Risk Factors and Scoring System for Predicting Bacterial Resistance to Cefepime as Used Empirically in Haematology Wards

**DOI:** 10.1155/2015/945769

**Published:** 2015-05-05

**Authors:** Hicham El Maaroufi, Agathe Goubard, Rabah Redjoul, Patrick Legrand, Cécile Pautas, Mohamed Mikdame, Kamal Doghmi, Andréa Toma, Sébastien Maury, Michael Schwarzinger, Catherine Cordonnier

**Affiliations:** ^1^Assistance Publique-Hôpitaux de Paris (APHP), Haematology Department, Henri Mondor Hospital and Paris-Est-Créteil University, 94000 Créteil, France; ^2^Haematology Department, Hôpital Militaire d'Instruction Mohamed V, Rabat, Morocco; ^3^Microbiology Laboratory, Henri Mondor Hospital, 94000 Créteil, France; ^4^Equipe ATIP/AVENIR, INSERM, UMR 738, 75018 Paris, France; ^5^University Paris Diderot, Sorbonne Paris Cité, UMR 738, 75018 Paris, France

## Abstract

*Objectives*. Bacterial resistance is of growing concern in haematology wards. As the inappropriate administration of empirical antibacterial may alter survival, we studied risk factors for resistance to our usual empirical first-line antibacterial therapy, cefepime. *Methods*. We retrospectively studied 103 first episodes of bacteraemia recorded in our haematology department over 2.5 years. Risk factors for cefepime-resistance were identified by multivariate logistic regression with backward selection (*P* < 0.05). A scoring system for predicting cefepime-resistance was built on independent factor, with an internal validation by the bootstrap resampling technique. *Results*. 38 (37%) episodes were due to Gram-negative bacteria. Fifty (49%) were due to bacteria resistant to cefepime. Cefepime resistance was significantly associated with a decreased survival at day 30 (*P* < 0.05). Three risk factors were independently associated with cefepime-resistance: acute lymphoblastic leukaemia; ≥18 days since hospital admission; and receipt of any *β*-lactam in the last month. Patients with ≥2 of these risk factors had a probability of 86% (CI 95%, 25 to 100%) to carry a cefepime-resistant strain. *Conclusion*. Using our scoring system should reduce the indication of very broad antibacterial regimens in the empirical, first-line treatment of febrile hematology patients in more than 80% of the cases.

## 1. Introduction

The empirical administration of a single antibiotic such as a cephalosporin of 4th generation or piperacillin-tazobactam is a standard of care in febrile neutropenic adult patients without severe sepsis or septic shock [1]. However, due to growing reports of bacterial resistance in haematology ward [2–8], the empirical administration of an antibiotic combination with the addition of aminoglycosides was recently recommended when bacterial resistance is suspected [1, 9] Accordingly, haematologists should be able to predict the level of risk of bacterial resistance in febrile neutropenic patients before deciding the empirical administration of one or more antibiotics. While several epidemiological studies identified risk factors for acquiring an extended-spectrum *β*-lactamase- (ESBL-) producing enterobacteria or other multidrug resistant bacteria in the community [3, 10, 11] or at hospital [12, 13], similar studies are scarce in haematology wards [3, 6, 14–17].

We conducted a retrospective study of the risk factors for bacterial resistance to cephalosporin as used empirically in our adult haematology department where the rate of ESBL is low. The objective of our study was to compute an easy-to-use scoring system for predicting bacterial resistance to cephalosporin and managing the first febrile episode in adult patients cared in haematology wards.

## 2. Design and Methods

### 2.1. Study Design

All consecutive episodes of bacteraemia recorded in our adult haematology department over 2.5 years were retrospectively assessed. The department comprises 26 single rooms for conventional hospitalization, including 18 laminar-air-flow rooms and a day clinic. The study was approved by the local ethics committee of Ile-de-France IX.

### 2.2. Inclusion Criteria, Exclusion Criteria, and Data Collection

We included all episodes of bacteraemia identified by the microbiology laboratory for a patient hospitalized in the haematology department during the study period. An episode of bacteraemia was defined either by an interval of at least 4 weeks between 2 series of blood cultures with the same pathogen (same species with the same antibiotic susceptibility pattern) or by blood culture(s) with a different pathogen at least more than 3 days of the previous one. In patients with ≥2 episodes of bacteraemia, we selectively included the first episode of bacteraemia as antibacterial management of consequent episodes usually depends on the first episode outcomes.

Microbiological data were collected from the laboratory chart. Medical charts were reviewed to record baseline characteristics of the patients, and any possible risk factor for bacterial resistance in the month preceding hospitalization and at onset of bacteraemia, including total length of stay at hospital, invasive procedures, and receipt (≥48 h) of antibiotic therapy with *β*-lactams. Mortality was assessed at day 30 after the episode of bacteraemia and at last follow-up.

### 2.3. Clinical Definitions

Fever and neutropenia were defined by a temperature ≥38.3°C, or ≥38°C twice at 8 h interval, and neutrophil counts <0.5 × 10^9^/L, respectively. Severe sepsis and septic shock were defined according to usual criteria [18].

### 2.4. Empirical Management at Fever Onset

According to our written protocol, any febrile neutropenic patient was clinically examined, a chest X-ray was performed, blood and urine samples were collected, and then a *β*-lactam antibiotic was administered within 4 hours after fever onset. Cefepime (CFP) is the antibiotic of choice for most febrile neutropenic episodes in our department [19]. Exceptions involve (1) cefotaxime for new patients hospitalized for less than 5 days; (2) piperacillin-tazobactam combination for patients with perineal cellulitis or suspicion of intra-abdominal infection; (3) the addition of glycopeptides for patients with severe sepsis or septic shock, severe mucositis, skin infection, or a previous infection with a methicillin-resistant* Staphylococcus aureus*; (4) the addition of aminoglycosides for patients with severe sepsis or septic shock [20]. For febrile patients without neutropenia, there is no written protocol in the ward, and patients were managed according to clinical presentation and the severity of immune depression. In all patients, antibiotic therapy was reevaluated at 48–72 h of fever onset with microbiological data. No colonization screening was systematically performed, except for patients coming from an intensive care unit or a foreign country. Patients receiving high-dose chemotherapy for acute leukaemia or a myeloablative conditioning regimen for allogeneic stem cell transplantation were given gut decontamination with oral colistin, gentamicin, and oral suspension of amphotericin B for the duration of neutropenia. In those, control of gut colonization was assessed weekly by stool cultures. Quinolone prophylaxis was not used.

### 2.5. Microbiological Methods

#### 2.5.1. Bacteraemia

At fever onset, 2 blood samples were collected at 1 h interval from the central intravenous line; otherwise, at least one blood sample was collected from a peripheral vein. Blood samples were processed by an automated blood system for detection of bacteraemia, the BacT/ALERT system (bioMérieux, Marcy l'Etoile, France), with an incubation of five days [21]. Bacterial isolates were identified by routine phenotypical tests using the API system (bioMérieux). When phenotypical testing failed to identify bacterial isolates, a 16S rRNA gene sequencing was performed as previously described [22] because of its particularly good accuracy for species designation. Bacteraemia was defined as ≥1 positive blood culture for bacteria other than coagulase-negative* Staphylococcus* (CNS),* Micrococcus*,* Corynebacterium* (other than* jeikeium* species), and* Bacillus *spp., for which ≥2 positive blood cultures were required.

#### 2.5.2. Bacterial Resistance to Cefepime (CFP-R)

Antibiotic susceptibility of bacterial isolates was performed by disk diffusion method (Biorad, Hercules, United States) according to the guidelines of the Comité de l'Antibiogramme de la Société Française de Microbiologie (CASFM, http://www.sfm-microbiologie.org/) which approved the MIC breakpoints of EUCAST for all antibiotics [23]. CFP-R was also defined according to CASFM guidelines or resulted from natural resistance (e.g., enterococci). Isolates with intermediate susceptibility were considered resistant. Streptococci resistance to aminoglycosides was defined by high level resistance without synergy with *β*-lactams. ESBL production was confirmed by double-disk synergy test [24] which shows a synergistic effect between clavulanate and third generation cephalosporins. Results were interpreted according to CASFM guidelines.

### 2.6. Statistical Analysis

To identify risk factors for CFP-R at bacteraemia onset, continuous variables were dichotomized with reference to the threshold maximizing the Youden Index (sensitivity plus specificity minus one), and univariate analyses were carried out using the chi-square test or exact Fisher test. All variables significant at *P* < 0.15 in univariate analyses were entered into a multivariate logistic regression with backward selection (*P* < 0.05 to stay) [25]. Possible two-way interaction effects were assessed using backward selection (*P* < 0.05 to stay) on the final model.

To predict the risk of CFP-R at fever onset, we developed two predictive binary scores based on the variables selected in multivariate analysis: (1) a score minimizing the number of false-negative diagnoses (i.e., high sensitivity and negative predictive value) taking the value of 1 when at least one criterion was positive, and 0 otherwise; (2) a score minimizing the number of false-positive diagnoses (i.e., high specificity and positive predictive value) taking the value of 1 when at least two criteria were positive, and 0 otherwise. Internal validation of the algorithm was performed by the bootstrap resampling technique [26]. Five hundred samples were drawn with replacement from the original data set, of the same size and event probability as the original data set. The predictive performance was assessed by the median (95% confidence interval) of the following indicators: (1) area under the receiver operating characteristic curve (AUROC) with a value of 1.0 indicating perfect prediction; (2) specificity, sensitivity, and positive and negative predictive values of the two predictive binary scores.

Survival in patients was calculated from bacteraemia onset with or without CFP-R strain by use of the Kaplan-Meier method and Logrank statistics. All analyses were based on two-sided *P* values, with *P* < 0.05 considered to indicate statistical significance. All analyses were carried out using SAS 9.2 statistical software (SAS Institute, Cary, NC).

## 3. Results

### 3.1. Patients and First Episode of Bacteraemia

The microbiology laboratory identified 155 episodes of bacteraemia for 103 patients cared at the haematology department during the study period. One or more episodes occurred in 33 patients with a median time interval of 8 days from the first to the second episode. We selected the first episode of bacteraemia for all 103 patients. The main characteristics of patients and first episodes of bacteraemia are summarized in Table 1. Most patients had acute leukaemia (81%), had received chemotherapy in the last 2 months (89%), and had a central intravenous catheter (83%) at onset of bacteraemia. Almost all (95%) patients were febrile, and most patients were deeply neutropenic (neutrophils < 100/mm^3^) (60%).

As first-line empirical antibiotic therapy, patients did receive cefepime (65%), cefotaxime (11%), piperacillin-tazobactam (19%), or imipenem (4%). Other carbapenems were not used. Overall, 54 (52%) patients did receive a cephalosporin as a single antibiotic, without difference between neutropenic and nonneutropenic patients (*P* = 0.24). In 25 (24%) patients including 11 severe sepsis and 2 septic shocks, cephalosporin was combined with one or more antibiotics (10 aminoglycosides; 10 glycopeptides; 2 quinolones; 3 aminoglycosides plus glycopeptides).

### 3.2. Microbiological Documentation of the Episode of Bacteraemia (Table 2)

The first episode of bacteraemia was due to Gram-positive bacteria in 65 (63%) and Gram-negative bacteria in 38 (37%). None of the 7* S. aureus* but 89% of the 28 CNS identified were methicillin-resistant. Among 14 oral streptococci, none was resistant to amoxicillin or gentamicin. Among 11 enterococci, none was resistant to vancomycin, 1 was resistant to amoxicillin, and 7 to gentamicin. Among 21 enterobacteria, 3 were ESBL-producers, 9 were resistant to cefotaxime, 1 was resistant to amikacin, and 1 was resistant to carbapenems. Among 4* P. aeruginosa*, none were resistant to aminoglycosides, piperacillin-tazobactam, or imipenem, 1 was resistant to ceftazidime, and 2 to fluoroquinolones.

The overall rate of CFP-R was 49% (CI 95%: 39% to 59%), significantly more frequent (*P* < 0.05) in the Gram-positive (57%) than in the Gram-negative (34%) episodes.

### 3.3. Risk Factors for Cefepime-Resistant Bacteraemia

In univariate analyses, neutropenia, steroids, other immunosuppressive therapies, gut decontamination, severe sepsis, and septic shock were not associated with CFP-R bacteraemia (Table 1). CFP-R bacteraemia was significantly associated with acute lymphoblastic leukaemia (ALL) (*P* < 0.05), having received *β*-lactams in the previous month (*P* < 0.01) or receiving *β*-lactams at fever onset (*P* < 0.001), and a longer time elapsed since hospital admission (*P* < 0.01) or central intravenous catheter insertion (*P* < 0.05). In multivariate analysis (Table 3), 3 risk factors remained significantly associated with CFP-R: ALL (OR = 6.0; *P* < 0.05); ≥18 days since hospital admission (OR = 4.7; *P* < 0.01); and having received any *β*-lactams at any time in the last month (OR = 3.6; *P* < 0.05).

### 3.4. Scoring System for Predicting Cefepime-Resistance

A scoring system was built on the three independent risk factors found in multivariate analysis (Table 4). As compared to patients without risk factors (score = 0), patients with one out of 3 risk factors (score = 1) or at least 2 risk factors (score = 2) had an odds-ratio of carrying a CFP-R strain of 4.6 (CI 95%, 1.8–11.9) and 21.3 (CI 95%, 4.4–104.1), respectively. We assessed the predictive performance of the scoring algorithm for predicting cefepime-resistance and found satisfying performance (median value of AUROC of 0.76; CI 95%, 0.67–0.84). A patient without risk factors had a probability of 27% to carry a CFP-susceptible strain (Table 4), while a patient with 2 or more risk factor had a probability of 86 to 100% (CI 95%, 25% to 100%) to carry a CFP-R strain.

### 3.5. Overall Survival from the First Episode of Bacteraemia

**Figure 1:**
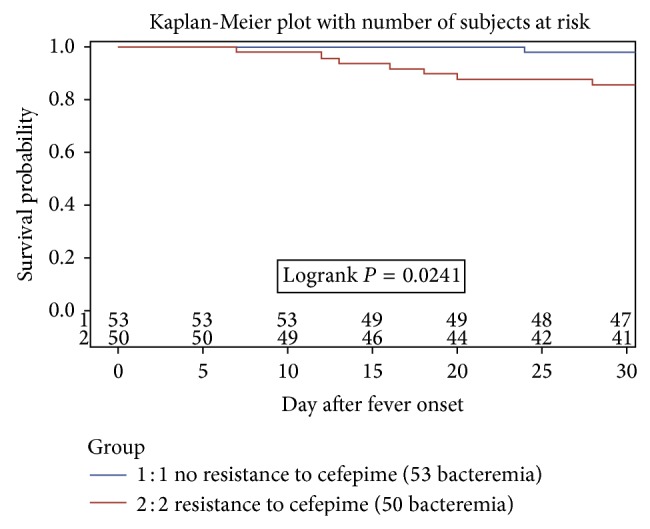
Impact of the susceptibility to cefepime on survival at day 30 after bacteraemia.

The Kaplan-Meier estimation of survival from the first episode of bacteraemia showed a significantly better survival at day 30 for the CFP-S group when compared to the CFP-R group (52/53: 98% and 43/50: 86%, resp., *P* < 0.05) (Figure 1). However, the difference in survival was no more significant at day 60 (49/53: 92% and 41/50, 82%, resp., *P* = 0.12) and at end of follow-up (median: 12 months; *P* = 0.66). After exclusion of 28 CNS, survival was significantly better at day 30 (49/50: 98% and 20/25: 80%, resp., *P* < 0.01) and day 60 (46/50: 92% and 19/25: 76%, resp., *P* < 0.05) for the CFP-S group when compared to the CFP-R group.

## 4. Discussion

Our study identified three risk factors for resistance to cefepime at the onset of a first episode of bacteraemia in haematology patients, mostly neutropenic. Moreover, CFP-R was significantly associated with early mortality. Among the factors we identified that for CFP-R previous administration of *β*-lactams was expected as already reported as a risk factor for later infection due to ESBL-producing strains in the community [10, 13, 27], in ICU [28, 29] or haematology patients [3]. Duration of hospitalization has also been shown to be an independent factor for acquiring a resistant bloodstream infection [27, 30], through prolonged exposure to hospital-acquired transmission. As for ALL as an independent risk factor for CFP-R, we have no univocal explanation. We do not use any specific prophylactic strategy in ALL in our center. When compared to the acute myeloid leukaemia patients (AML), ALL patients have the main particularities of multiple hospitalizations, longer treatments, and repeated courses of steroids which may mask fever. Therefore, we routinely perform blood cultures every other day during the steroid periods in order to detect asymptomatic bacteraemia. This may explain the onset of more CFP-R bacteraemia in ALL when compared to AML patients who benefit from blood cultures only when symptomatic. However, only 3 of our 13 ALL patients were not febrile at time of bacteraemia. The recognition of ALL as a high-risk population for bacterial resistance in the haematology ward should encourage performing systematic blood cultures even when afebrile under steroids.

Due to increasing rates of bacterial resistance worldwide and the paucity of new antibacterial classes, warnings have been given in order to protect the antibiotics we have for the more threatened patients [31, 32]. Recent data on bacterial resistance are available for onco-haematology patients who cumulate antibacterial courses, invasive devices, immune depression, and long hospital stays [7, 14, 15, 17, 30]. However, despite common factors in cancer patients, the epidemiologic situation is clearly different from one country to another [5]. As patients with resistance strains are more prone to receive an inappropriate first-line antibacterial therapy [3, 27, 33], the choice of the first line should be tailored to the individual risk, each time the risk can be anticipated. In high-risk neutropenic patients, the IDSA guidelines recommend the use of one of three antipseudomonal *β*-lactams-cefepime, a carbapenem (meropenem or imipenem-cilastatin), or piperacillin-tazobactam [1]. Even in the case of uncomplicated presentation, they also recommend to take in account previous infection or colonization with vancomycin-resistant enterococcus, methicillin-resistant* Staphylococcus aureus*, ESBL-producing bacteria, or carbapenemase-producing* Klebsiella* to reinforce empirical therapy until microbiological results. Similarly, the recent ECIL guidelines recommend a deescalation approach using upfront broad spectrum antibacterials, eventually including a carbapenem or combination, in patients who are colonized or had past history of infection with resistant pathogens, especially ESBL, producing strains, complicated infection or septic shock, or in centers with a high prevalence of resistance [9]. However, in the lack of previous colonization or infection, in centers with low levels of resistance as ours, and in the lack of severe clinical presentation, it may be difficult to identify patients who should benefit from a broader initial therapy. On the one hand, an unnecessary deescalation approach increases the costs of anti-infectives and the use of precious molecules. On the other hand, an escalation therapy is a more risky short-term option at the individual level. With an ESBL incidence <3% among all bacteraemia, and of 14% among enterobacteriae, much lower than the 22–44% reported from other recent European centers [5, 6, 8, 14], we can consider our local epidemiology as very favorable when compared to others in similar departments. Therefore, we were more interested in factors associated with resistance to our usual antibacterial first-line therapy, rather than in factors associated with ESBLs, and deliberately chose to develop a score combining both Gram-positive and Gram-negative resistant bacteria. This was driven by very practical considerations to improve the management of patients at bedside, as the most important for febrile neutropenia is to give an adequate antibacterial therapy during the first 48 hours, until microbiological documentation. The fact that half of the CFP-R strains were CNS does not, in our opinion, challenge the interest of this score since these CNS are usually treated in routine, and although the mortality of CNS bacteremia is low, they are often the only cause of fever in these patients. The international guidelines [1, 9] do not tell us what is the acceptable target rate of coverage for first-line empirical treatment of febrile neutropenic patients. In our opinion, coverage of at least 70% appears to be a very minimal requirement. Apart from CNS bacteraemia, only 24 (23%) of episodes in our study deserved a modification of our standard empirical therapy, 11 for enterococcal bacteraemia, and 13 for CFP-R Gram-negative bacteraemia. Using our score should allow avoiding missing the 25% of episodes at higher risk of resistance.

We recognize the limits of this work. First, we limit our study to first episodes of bacteraemia, considering that a first episode of bacteraemia could impact on the later episodes and that consequently most data would not have been explicative from the second episode. Second, we did not look at the impact of colonization which we do not routinely screen except on stool cultures in patients receiving gut decontamination. However, during the study period, we did not document any ESBL in the stools of these patients. Finally, we should notice that CFP-R was not associated with severe sepsis (17% of the episodes) or septic shock (4% of the episodes) in our experience, maybe due to the low number of patients of our series.

Following the results of our study and in addition to the guidelines for enlarging the antibacterial spectrum of initial therapy in case of complicated presentation or previous infection or colonization with resistant bacteria, we now consider enlarging the initial antibacterial spectrum in uncomplicated patients with a score ≥2 by adding amikacin, or using imipenem instead of cefepime for the first 48 h of treatment, till the microbiological results, and then deescalate each time possible. We clearly discourage the use of penems or combinations for patients with a score ≤1.

In conclusion, as in other populations, bacterial resistance in haematology wards is mostly healthcare related and influenced by previous antibiotic use and length of hospital stay. Specific populations such as patients with ALL should be identified at high risk. The modification of a standard, cephalosporin-based first-line therapy may be warranted in one-fourth of the cases by use of the simple score we propose. However, most of our patients have a score ≤1 and should be proposed an escalation strategy.

## Figures and Tables

**Table 1:** Baseline characteristics and clinical features at onset of the first episode of bacteraemia in the haematology ward.

Characteristic	All patients	Cefepime resistance	No cefepime resistance	*P* ^a^
	*n* = 103	*n* = 50	*n* = 53	
Patient characteristics				
Age, mean (SD), yr	53 (16)	51 (16)	54 (16)	0.32
Female, *n* (%)	45 (43)	23 (46)	22 (42)	0.65
Primary diagnosis, *n* (%)				
Acute myeloid leukaemia and myelodysplastic syndrome	71 (68)	30 (60)	41 (77)	
Acute lymphoblastic leukaemia^b^	13 (13)	11 (22)	2 (4)	0.005
Lymphoma or myeloma	13 (13)	5 (10)	8 (15)	
Nonmalignant haematological disease	6 (6)	4 (8)	2 (4)	
Phase of therapy, *n* (%)				
No chemotherapy in the previous 2 months	11 (11)	5 (10)	6 (11)	
Induction^c^	26 (25)	15 (30)	11 (21)	0.28
Relapse	20 (20)	9 (18)	11 (21)	
Consolidation	21 (20)	8 (16)	13 (24)	
Allogeneic Stem Cell Transplant	25 (24)	13 (26)	12 (23)	
Graft-versus-host disease, *n* (%)^d^	20 (80)	11 (85)	9 (75)	0.64
Comorbidities, *n* (%)^e^	9 (9)	5 (10)	4 (8)	0.66
Risk factors in the month preceding bacteraemia				
Transfer from abroad, *n* (%)	6 (6)	4 (8)	2 (4)	0.43
Intensive care unit, *n* (%)	3 (3)	3 (6)	0	0.11
Surgery, *n* (%)	3 (3)	2 (4)	1 (2)	0.61
Antibiotic therapy with betalactams	25 (24)	18 (36)	7 (13)	0.007
Risk factors at the onset of bacteraemia				
Neutrophil count, *n* (%)				
Neutrophils ≥500/mm^3^	27 (26)	13 (26)	14 (26)	0.56
Neutrophils 100–500/mm^3^	14 (14)	5 (10)	9 (17)	
Neutrophils <100/mm^3^	62 (60)	32 (64)	30 (57)	
Corticosteroid therapy, *n* (%)	15 (15)	9 (18)	6 (11)	0.34
Another immunosuppressive therapy, *n* (%)	23 (22)	13 (26)	10 (19)	0.39
Severe mucositis, *n* (%)	8 (8)	6 (12)	2 (4)	0.15
Gut decontamination, *n* (%)	76 (74)	39 (78)	37 (70)	0.35
Antibiotic therapy with *β*-lactams, *n* (%)	21 (20)	17 (34)	4 (19)	0.001
Time elapsed since hospital admission, mean (SD), days	15 (13)	19 (15)	12 (10)	0.003
Central venous catheter, *n* (%)	85 (83)	42 (84)	43 (81)	0.70
Time elapsed since central venous catheter insertion, mean (SD), days^f^	17 (15)	20 (19)	14 (11)	0.040
Urinary catheter, *n* (%)	1 (1)	1 (2)	0	0.49
Clinical features of bacteraemia				
Fever, *n* (%)	98 (95)	46 (92)	52 (98)	0.20
Severe sepsis, *n* (%)	17 (17)	6 (12)	11 (21)	0.23
Shock, *n* (%)	4 (4)	1 (2)	3 (6)	0.62
Unknown origin of bacteraemia, *n* (%)	59 (58)	31 (62)	28 (53)	0.35

^a^By chi-square test or Fisher's exact test for qualitative variables; by Wilcoxon rank-sum test for skewed quantitative variables.

^b^By chi-square test comparing acute lymphoblastic leukaemia with other situations.

^c^By chi-square test comparing induction with other situations.

^d^Estimates were computed for 25 patients with ASCT.

^e^2 chronic obstructive pulmonary disease; 2 chronic heart disease; 2 cirrhosis; 2 diabetes mellitus; 1 diabetes mellitus and cirrhosis.

^f^Estimates were computed for 85 patients with intravascular catheter.

**Table 2:** Microbial documentation of the 103 first episodes of bacteraemia in haematology patients and resistance to cefepime.

	Number (%)	Number (%) resistant to cefepime
Gram-positive cocci	65 (63)	37 (57)
Staphylococci		
*Staphylococcus aureus *	7	0 (0)
*Coagulase negative staphylococci *	28	25 (89)
Streptococci and enterococci		
*Oral streptococci *	14	0 (0)
*Enterococci* ^a^	11	11 (100)
*Other Gram-positive *	5	1 (20)
Gram-negative bacteria	38 (37)	13 (34)
*Enterobacteriae* ^b^	21	8 (38)
*Pseudomonas aeruginosa *	4	1 (25)
*Acinetobacter *sp.	6	1 (17)
*Nonaeruginosa pseudomonas *	3	0 (0)
*Stenotrophomonas *sp.	3	3 (100)
*Leptotrichia buccalis *	1	0 (0)

Total	103	50 (49%)

^a^None enterococci was resistant to vancomycin.

^a^Includes 3 ESBL-producing Enterobacteriaceae and 1 carbapenemase-producing *E coli*.

**Table 3:** Risk factors for cefepime resistance in multivariate logistic regression.

Risk factor	Unadjusted OR	Adjusted OR	*P*
Acute lymphoblastic leukaemia	7.2 (1.5 to 34.3)	6.0 (1.1 to 31.8)	0.036
Antibiotic therapy with *β*-lactams (current or in the last month)	4.8 (1.8 to 12.6)	3.6 (1.3 to 10.5)	0.017
Time elapsed since hospital admission ≥18 days	6.7 (2.4 to 18.4)	4.7 (1.6 to 13.9)	0.005
Time elapsed since central venous catheter insertion ≥16 days	2.8 (1.2 to 6.6)	—	

**Table 4:** Predictive score of cefepime resistance with 3 risk factors at onset of the first episode of bacteraemia^a^.

Score	Number (%) of patients	% cefepime resistance	OR
0 risk factor	55 (53)	27	1
1 risk factor	30 (29)	63	4.6 (1.8 to 11.9)
2 risk factors	14 (14)	86	21.3 (4.4 to 104.1)
3 (all) risk factors	4 (4)	100	

^a^The 3 criteria are acute lymphoblastic leukaemia, beta-lactams administration during the month or at onset of bacteraemia, and time elapsed since hospital admission >18 days.
